# Familial aortic disease and a large duplication in chromosome 16p13.1

**DOI:** 10.1002/mgg3.371

**Published:** 2018-02-14

**Authors:** Philipp Erhart, Tobias Brandt, Beate K. Straub, Ingrid Hausser, Sabine Hentze, Dittmar Böckler, Caspar Grond‐Ginsbach

**Affiliations:** ^1^ Department of Vascular and Endovascular Surgery University Hospital Heidelberg Heidelberg Germany; ^2^ Suva/Swiss National Accident Insurance Fund Lucerne Switzerland; ^3^ Department of Pathology University Hospital Heidelberg Heidelberg Germany; ^4^ Department of Pathology University Medicine Mainz Mainz Germany; ^5^ Human genetical practice Heidelberg Germany; ^6^ Department of Neurology University Hospital Heidelberg Heidelberg Germany

**Keywords:** cosegregation, duplication 16p13.1, familial thoracic aortic aneurysm and dissection, whole exome sequencing

## Abstract

**Background and purpose:**

A recurrent duplication of chromosome 16p13.1 was associated with aortic dissection as well as with cervical artery dissection. We explore the segregation of this duplication in a family with familial aortic disease.

**Methods:**

Whole exome sequencing (WES) analysis was performed in a patient with a family history of aortic diseases and ischemic stroke due to an aortic dissection extending into both carotid arteries.

**Results:**

The index patient, his affected father, and an affected sister of his father carried a large duplication of region 16p13.1, which was also verified by quantitative PCR. The duplication was also found in clinically asymptomatic sister of the index patient. WES did not detect pathogenic variants in a predefined panel of 11 genes associated with aortic disease, but identified rare deleterious variants in 14 genes that cosegregated with the aortic phenotype.

**Conclusions:**

The cosegregation of duplication 16p13.1 with the aortic phenotype in this family suggested a causal relationship between the duplication and aortic disease. Variants in known candidate genes were excluded as disease‐causing in this family, but cosegregating variants in other genes might modify the contribution of duplication 16p13.1 on aortic disease.

## 1 BACKGROUND

Large duplications of a region of chromosome 16p13.1 containing at least nine protein‐coding genes (*MPV17L, C16orf45, KIAA0430, NDE1, MYH11, C16orf63, ABCC1, ABCC6, NOMO3*) occur in the European population at a low frequency of about 0.1% (Grozeva et al., 2012). Significant enrichment of 16p13.1 duplications was reported in patients with aortic dissections or with cervical artery dissections (Kuang et al., 2011; Grond‐Ginsbach, Chen, et al., 2017), which suggests that carriers of the duplication have an increased risk for arterial dissection. However, in two pedigrees with familial thoracic aneurysms and dissections, the 16p13.1 duplication did not cosegregate with the aortic phenotype (Kuang et al., 2011).

In this study, we analyzed a family with familial thoracic aortic aneurysms and dissections (FTAAD) and 16p13.1 duplication. We tested for cosegregation of the duplication and the arterial phenotype and searched for additional genetic risk factors by whole exome sequencing analysis (WES).

## 2 PATIENTS AND METHODS

**Figure 1:**
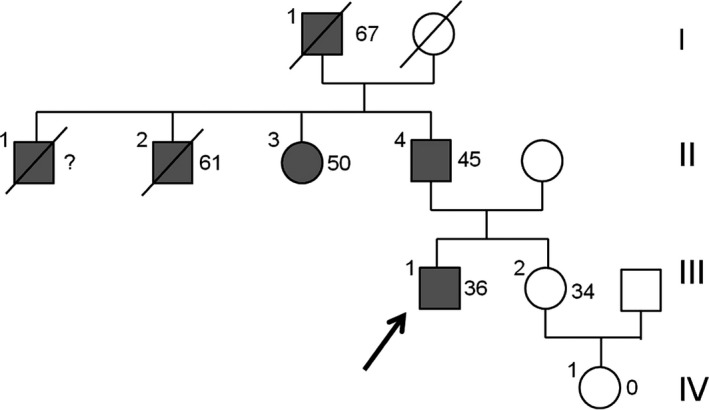
Four‐generation pedigree of family with familial thoracic aortic aneurysms and dissection. Arrow points to the index patient. Filled symbols indicate patients affected with aortic disease and age of onset of first symptoms. For unaffected relatives, age at genetic testing is indicated. Molecular testing results: III‐1 (index patient): Dupl. 16p13.3 (*LTBP4*,* Gly1553Ser variant)*, II‐3: 16p13.3 (*LTBP4*,* Gly1553Se variant)*, II‐4: Dupl. 16p13.3 (*LTBP4*,* Gly1553Ser variant)*, III‐2: Dupl. 16p13.3 (*LTBP4*,* Gly1553Ser negative)*

The nonsmoking, physically active index patient of this study (patient III‐1 indicated by an arrow in Figure 1) suffered aortic dissection, extending into both carotid arteries causing ischemic stroke at an age of 36 years. The affected aortic root could be replaced successfully by open surgery (modified Bentall operation) and neurological rehabilitation was initiated after clinical recovery.

The patient reported a family history of aortic disease: (1) His father who is a heavy smoker (patient II‐4) had an acute aortic dissection at an age of 45 years; (2) his grandfather (patient I‐1) suffered a ruptured abdominal aneurysm and died at an age of 67; (3) his paternal aunt (patient II‐3) suffered aortic dissection Type A at an age of 50 years; and his uncle, hypertensive since adulthood (patient II‐2) died from aortic rupture at an age of 61 years. A third uncle (patient II‐1) died at an early age from aortic disease. Unfortunately, we could not validate the diagnosis of this latter relative by official clinical records. Genetic analysis of the family was initiated as the healthy sister of the index patients (subject III‐2 in Figure 1) asked for genetic advice concerning her risk for aortic disease during pregnancy.

Peripheral blood from subjects III‐1, III‐2, II‐3, and II‐4 was used for DNA extraction. Exome sequencing was performed at the German Research Center for Environmental Health, Helmholtz Zentrum München, on a Genome Analyzer IIx system (Illumina) after in‐solution enrichment of exonic sequences (SureSelect Human All Exon 38 Mb kit, Agilent). Read alignment to the human genome assembly hg19 was performed with the Burrows‐Wheeler Aligner (BWA) software, version 0.5.8. Single‐nucleotide variants (SNV) were detected with SAMtools (v 0.1.7), as described (Grond‐Ginsbach, Brandt, et al., 2017).

SNV findings were prioritized if (1) their coverage (depth) was ≥40 reads; (2) they caused nonsense or stop‐loss substitutions in the encoded gene product, or missense substitutions with polyphen‐2 probability scores ≥0.95 (Adzhubei et al., 2010); and (3) they were found in less than 1,000 of 10,314 analyzed in‐house samples. In a subsequent step, nonsense variants with SIFT (Sorting Intolerant From Tolerant) scores ≥0.05 were rejected (Ng & Henikoff, 2003). In a final filtering step, variants occurring at a frequency >0.01 in the European (non‐Finnish) populations from the ExAC database (http://exac.broadinstitute.org) were removed (Lek et al., 2016).

Postanalytic interrogation of prioritized findings started with the analysis of a predefined panel of 11 candidate genes (*ACTA2, MYH11, FBN1, COL3A1, COL4A1, TGFBR1, TGFBR2, TGFB2, SMAD3, MYLK, SLC2A10*) associated with arterial connective tissue disorders (vascular Ehlers‐Danlos syndrome, Marfan syndrome, Loeys‐Dietz syndrome, familial thoracic aortic aneurysms and dissections, arterial tortuosity syndrome; Grond‐Ginsbach, Brandt, et al., 2017; Bowdin, Laberge, Verstraeten, & Loeys, 2016), followed by the exploration of all prioritized variants in genes associated with the transforming growth factor beta receptor (TGFBR)‐signaling pathway as identified from the GeneOntology (GO) database (term GO:0007179) (Watson, Laskowski, & Thornton, 2005).

The detection of a large duplication on chromosome 16p13.1 was confirmed by quantitative evaluation of a restriction fragment length polymorphism (RFLP) assay for three SNPs within the duplication (*rs4985155, rs2075511,* and *11075290*). Comparison of the fluorescence intensities of the allele‐specific bands of family members and of control samples enabled the detection of alleles with an additional copy as digested PCR fragments of double intensity. All tested family members were heterozygous for these variants.

The aortic resection specimen was routinely formalin‐fixed and paraffin‐embedded (FFPE), cut and stained with hematoxylin‐eosin. In addition, the FFPE‐material was reprocessed for ultrastructural analysis by transmission electron microscopy and analyzed with a JEM 1400 (JEOL, Akishima, Japan).

## 3 RESULTS

Figure 1 shows the pedigree of the index patients (indicated by an arrow) with ischemic stroke due to thoracic aortic dissection (Stanford Type A) extending into both carotid arteries. The sister of the patients (subject III‐2) asked for genetic advice about her risk for aortic events during pregnancy.

DNA samples from the index patient (III‐1) and his aunt (II‐3) were analyzed by whole exome sequencing (WES). Variants found in both subjects were prioritized according to functionality and frequency as described in detail in the Methods section. Fourteen rare variants were prioritized as rare and probably deleterious (Polyphen‐2 probability score >0.95) in the family. None of the prioritized variants affected a gene that was associated with aortic disease (Table 1).

**Table 1:** Prioritized variants found in both analyzed patients (II‐3 and III‐1). dbSNP = identified or the variant in the NCBI (National Center for Biotechnology Information) database of short genetic variants (https://www.ncbi.nlm.nih.gov/projects/SNP/); ExAC = Frequency of the variant in the European (non‐Finnish) populations from the exome aggregation consortium database (http://exac.broadinstitute.org); OMIM = Online Mendelian Inheritance in Man database (https://www.omim.org). MalaCards = the Human disease database (http://www.malacards.org). AR = autosomal recessive, AD = autosomal dominant

Gene	dbSNP	Polyphen	ExAC	Association (OMIM/MalaCards)
STON2, p. Thr532Ala	Not found	0.997	0	Schizophrenia
MIEN1, p. Arg94Stop	rs559490756		0	
TSPAN10, frameshift	Not found		0	
NPHS1, frameshift	Not found		0	Nephrotic syndrome, type 1, AR
EOGT, p. Met246Ile	Not found	0.986	0	Adams‐Oliver syndrome 4, AR
FRAS1, p. Cys575Tyr	Not found	0.999	0	Fraser syndrome, AR
BRD8, frameshift	Not found		0	
LTBP4, p. Gly1553Ser	rs376792458	0.999	1/15442	Cutis laxa, type IC, AR
CWC25, p. Arg287Leu	rs199507186	0.972	35/65648	
C2orf88, p. Gly2Asp	rs200124996	1	38/66478	
MMP10, p. Tyr400Stop	rs147267769		297/121274	
RYR1, p. Arg1679His	rs146504767	0.999	171/65042	Central core disease, AR, AD
CD52, p. Thr10Asn	rs77928789	0.982	211/66740	
ACSM5, p. Arg72His	rs149315908	0.974	302/65770	

The increased number of reads and unequal frequencies of bi‐allelic variants (1/3 vs. 2/3) in genomic region *16:14,916,662‐16,306,102* suggested the presence of a large duplication of chromosome region 16p13.3 in both patients. By quantitative RFLP analysis, the presence of the duplication was confirmed in the index patient, his aunt, his father (patient II‐4 in Figure 1) and his nonaffected sister (III‐2). The observed duplication was mapped on the human genome and found to cover nine protein‐coding genes, including *MYH11* (encoding the smooth muscle cell‐specific myosin heavy chain 11) and *ABCC6* (encoding the ATP‐binding cassette subfamily C member 6).

The *MYH11* gene as well as the *LTBP4* gene were associated with GeneOntology term “transforming growth factor beta 5 receptor signaling pathway signaling *GO:0007179*). We tested for the presence of the *LTBP4*,* Gly1553Ser* variant in all relatives with DNA available. The *LTBP4* variant was found in all affected relatives, but was absent in the healthy sister (III‐2) of the index patient.

**Figure 2:**
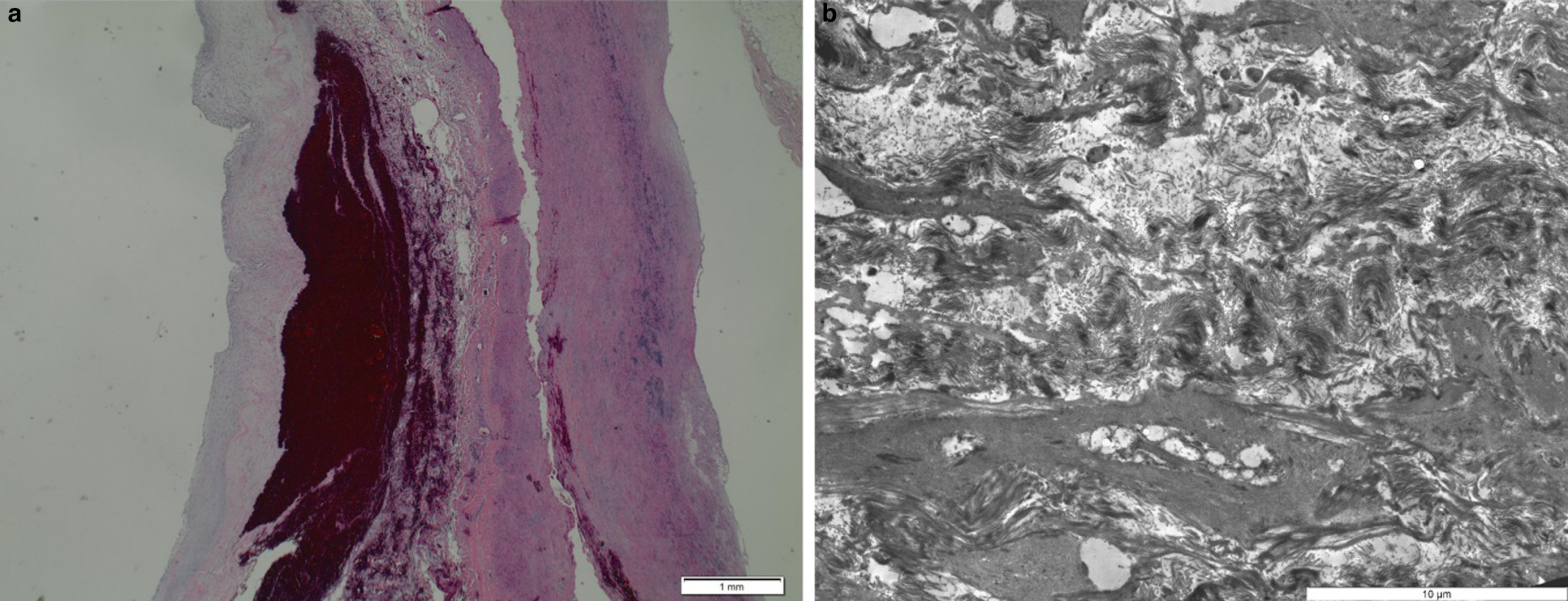
Histology and electron microscopy of the aorta of patient III‐1. Left: hematoxylin‐stained section of the aorta showing dissection and hemorrhage as well as myxoid media degeneration. On the left: ultrastructure of the aorta with smooth muscle cell degeneration, but without characteristic morphologic features of Ehlers‐Danlos‐syndrome, Marfan‐syndrome, or a heritable connective tissue disorder with known ultrastructural aberrations

In addition, the histology of the index patient was analyzed, showing aortic dissection and mucoid media degeneration as well as slight arteriosclerotic changes. To gain further insight into the underlying cause of aortic dissection, the FFPE‐material was processed for transmission electron microscopy (Figure 2). Ultrastructure of perilesional aortic tissue did not show any specific changes, especially no alterations of collagen or elastic fibers, so no typical alterations as seen in Marfan Syndrome or Ehlers‐Danlos‐Syndrome type 4, no specific alterations in smooth muscle cells were seen, especially no alterations in myofibrils or focal adhesions of smooth myocytes as seen in inherited connective tissue diseases.

## 4 DISCUSSION

In this study, we present a family with familial aortic aneurysms and dissections. DNA from the index patients, two affected relatives, and from one unaffected relative was available for molecular analysis. No DNA was available from two further relatives with fatal aortic rupture.

Key finding of this study is the cosegregation of aortic disease with a large duplication in chromosome 16p13.1. In earlier studies, this duplication was associated with an increased risk of aortic dissection and of cervical artery dissection.

The clinically asymptomatic sister of the index patient was also carrying the 16p13.1 duplication. This finding may predict that this woman of age 34 years could develop aortic events in the future. Some studies of familial vascular diseases therefore differentiate disease phenotypes as definite versus possible or consider “affected cases” only.

In an earlier report, however, the 16p13.1 duplication did not cosegregate with aortic disease in two affected families. Hence, the detection of 16p13.1 duplication in the clinical asymptomatic sister of the index patient does not necessarily imply that she is at high risk of aortic disease. A cesarean section was performed to avoid excess pressure during childbirth. We hypothesize that aortic disease has a multifactorial pathogenesis, favored by duplication 16p13 as well as by additional risk factors. As the pathogenic role of duplication 16p13.1 in aortic disease is possibly related to disruption of the *TGF‐beta* pathway (Kuang et al., 2011; Kwartler et al., 2014), the finding of a second genetic variant (*LTBP4*) affecting the *TGF‐beta* receptor signaling pathway in all affected relatives may suggest a digenic inheritance of the aortic phenotype in this family. The *LTBP4* variant was not found in the healthy sister of the index patient. From histology and electron microscopy of the index patient, signs of degeneration and of dissection of the aorta were observed, but no specific changes were detected. Especially, as only perilesional aortic tissue from the dissected area was available, we could not detect significant alterations in smooth muscle cells, especially no alterations in myofibrils or focal adhesions of myocytes suspicious for inherited connective tissues diseases.

This report of a patient with aortic disease and a family history of aortic disease showed cosegregation of duplication 16p13.1 with the aortic phenotype and hypothesized that a second rare variant affecting the *TGF‐beta* signaling pathway may have amplified the impact of duplication 16p13.1.

The pathogenicity of the *MYH11* duplication in the presented family is not proven, in spite of suggestive evidence for an association of this duplication with arterial dissection (Kuang et al., 2011; Grond‐Ginsbach, Chen, et al., 2017). The observation that the duplication at 16p13.1 encompasses four ohnologs (*NDE1, MYH11, ABCC1,* and *ABCC6)* further suggests its pathogenicity, because ohnologs were shown to be dose‐sensitive and overrepresented in pathogenic copy number variation (Makino & McLysaght, 2010; McLysaght et al., 2014; Tropeano et al., 2013).

Genetic causes of aortic dissections should be suspected in young patients with a positive familial history for cardiovascular events. Human genetic guidance and definitive treatment should be performed at highly specialized centers to achieve best familial counseling and clinical success.

## CONFLICT OF INTEREST

The author and co‐authors declare to have no conflict of interests.
